# Gene model for the ortholog of *Ilp3* in *Drosophila ananassae*

**DOI:** 10.17912/micropub.biology.000958

**Published:** 2025-06-04

**Authors:** Madeline L. Gruys, James O’Brien, Alyssa C. Koehler, Alejandro Almazan, Katheryn Opperman, Rachel Sterne-Marr, Zeynep Ozsoy, Maire Kate Sustacek, Jacqueline Wittke-Thompson, Andrew M Arsham, Stephanie Toering Peters, Chinmay P. Rele, Laura K Reed

**Affiliations:** 1 The University of Alabama, Tuscaloosa, AL USA; 2 Oklahoma Christian University, Edmond, OK, USA; 3 University of St. Francis, Joliet, IL USA; 4 Wartburg College, Waverly, IA USA; 5 Siena College, Loudonville, NY USA; 6 Colorado Mesa University, Grand Junction, CO, USA; 7 Minneapolis Community and Technical College, Minneapolis, MN USA; 8 Bemidji State University, Bemidji, MN

## Abstract

Gene model for the ortholog of *Insulin-like peptide 3* ( *Ilp3* ) in the May 2011 (Agencourt dana_caf1/DanaCAF1) Genome Assembly (GenBank Accession: GCA_000005115.1 ) of *Drosophila ananassae* . This ortholog was characterized as part of a developing dataset to study the evolution of the Insulin/insulin-like growth factor signaling pathway (IIS) across the genus *Drosophila* using the Genomics Education Partnership gene annotation protocol for Course-based Undergraduate Research Experiences.

**Figure 1. Genomic neighborhood and gene model for Ilp3 in Drosophila ananassae :**
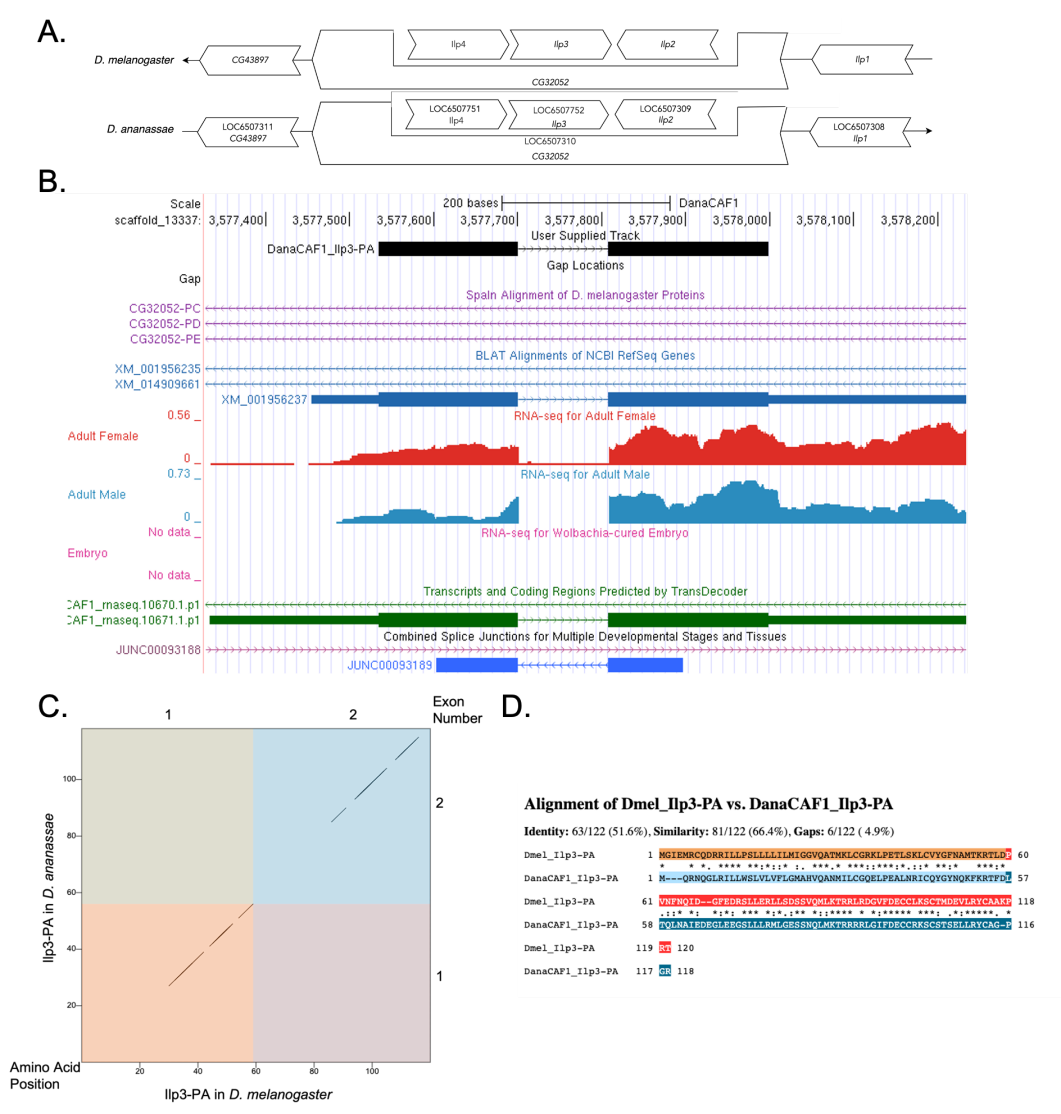
**
(A) Synteny comparison of the genomic neighborhoods for *Ilp3* in *Drosophila melanogaster* and *D. ananassae* .** Thin underlying arrows indicate the DNA strand within which the reference gene– *Ilp3* –is located in *D. melanogaster* (top) and *D. ananassae* (bottom). The thin arrow pointing to the right indicates that *Ilp3* is on the positive (+) strand in *D. ananassae* , and the thin arrow pointing to the left indicates that *Ilp3* is on the negative (-) strand in *D. melanogaster* . The wide gene arrows pointing in the same direction as *Ilp3* are on the same strand relative to the thin underlying arrows, while wide gene arrows pointing in the opposite direction of *Ilp3* are on the opposite strand relative to the thin underlying arrows. White gene arrows in *D. ananassae* indicate orthology to the corresponding gene in *D. melanogaster* . Gene symbols given in the *D. ananassae* gene arrows indicate the orthologous gene in *D. melanogaster* , while the locus identifiers are specific to *D. ananassae* . **(B) Gene Model in GEP UCSC Track Data Hub** (Raney et al., 2014). The coding-regions of *Ilp3* in *D. ananassae* are displayed in the User Supplied Track (black); coding CDSs are depicted by thick rectangles and introns by thin lines with arrows indicating the direction of transcription. Subsequent evidence tracks include BLAT Alignments of NCBI RefSeq Genes (dark blue, alignment of Ref-Seq genes for *D. ananassae* ), Spaln of *D. melanogaster* Proteins (purple, alignment of Ref-Seq proteins from *D. melanogaster* ), Transcripts and Coding Regions Predicted by TransDecoder (dark green), RNA-Seq from Adult Females and Adult Males (red and light blue, respectively; alignment of Illumina RNA-Seq reads from *D. ananassae* ), and Splice Junctions Predicted by regtools using *D. ananassae* RNA-Seq ( SRP006203 , SRP007906 , PRJNA257286 , PRJNA388952 ). Splice junctions shown have a minimum read-depth of 10 with 10-49, 50-99, 100-499, 500-999, and >1000 supporting reads in blue, green, pink, brown, and red, respectively. **(C) Dot Plot of Ilp3-PA in *D. melanogaster* ( *x* -axis) vs. the orthologous peptide in *D. ananassae* ( *y* -axis).** Amino acid number is indicated along the left and bottom; coding-CDS number is indicated along the top and right, and CDSs are also highlighted with alternating colors. Line breaks in the dot plot indicate mismatching amino acids at the specified location between species. The line breaks shown are small and determined to be insignificant in the determination of the putative ortholog of *Ilp3* in *D. ananassae* . **(D) Protein alignment between *D. melanogaster* Ilp3-PA and its putative ortholog in *D. ananassae* .** The alternating colored rectangles represent adjacent CDSs. The symbols in the match line denote the level of similarity between the aligned residues. An asterisk (*) indicates that the aligned residues are identical. A colon (:) indicates the aligned residues have highly similar chemical properties—roughly equivalent to scoring > 0.5 in the Gonnet PAM 250 matrix (Gonnet et al., 1992). A period (.) indicates that the aligned residues have weakly similar chemically properties—roughly equivalent to scoring > 0 and ≤ 0.5 in the Gonnet PAM 250 matrix. A space indicates a gap or mismatch when the aligned residues have a complete lack of similarity—roughly equivalent to scoring ≤ 0 in the Gonnet PAM 250 matrix.

## Description

**Table:** 

* This article reports a predicted gene model generated by undergraduate work using a structured gene model annotation protocol defined by the Genomics Education Partnership (GEP; thegep.org ) for Course-based Undergraduate Research Experience (CURE). The following information in this box may be repeated in other articles submitted by participants using the same GEP CURE protocol for annotating Drosophila species orthologs of Drosophila melanogaster genes in the insulin signaling pathway. * "In this GEP CURE protocol students use web-based tools to manually annotate genes in non-model *Drosophila* species based on orthology to genes in the well-annotated model organism fruitfly *Drosophila melanogaster* . The GEP uses web-based tools to allow undergraduates to participate in course-based research by generating manual annotations of genes in non-model species (Rele et al., 2023). Computational-based gene predictions in any organism are often improved by careful manual annotation and curation, allowing for more accurate analyses of gene and genome evolution (Mudge and Harrow 2016; Tello-Ruiz et al., 2019). These models of orthologous genes across species, such as the one presented here, then provide a reliable basis for further evolutionary genomic analyses when made available to the scientific community.” (Myers et al., 2024). “The particular gene ortholog described here was characterized as part of a developing dataset to study the evolution of the Insulin/insulin-like growth factor signaling pathway (IIS) across the genus *Drosophila* . The Insulin/insulin-like growth factor signaling pathway (IIS) is a highly conserved signaling pathway in animals and is central to mediating organismal responses to nutrients (Hietakangas and Cohen 2009; Grewal 2009).” (Myers et al., 2024). “ *D* . *ananassae* (NCBI:txid7217) is part of the *melanogaster* species group within the subgenus *Sophophora* of the genus *Drosophila* (Sturtevant 1939; Bock and Wheeler 1972). It was first described by Doeschall (1858). *D. ananassae* is circumtropical (Markow and O'Grady 2005; https://www.taxodros.uzh.ch, accessed 1 Feb 2023), and often associated with human settlement (Singh 2010). It has been extensively studied as a model for its cytogenetic and genetic characteristics, and in experimental evolution (Kikkawa 1938; Singh and Yadav 2015).” (Lawson et al., 2024).

We propose a gene model for the *D. ananassae* ortholog of the *D. melanogaster* *Insulin-like peptide 3* ( *Ilp3* ) gene. The genomic region of the ortholog corresponds to the uncharacterized protein XP_001956273.1 (Locus ID LOC6507752 ) in the *D. ananassae* May 2011 (Agencourt dana_caf1/DanaCAF1 Genome Assembly ( GCA_000005115.1 ; Drosophila 12 Genomes Consortium et al., 2007)). This model is based on RNA-Seq data from *D. ananassae* ( SRP006203 , SRP007906 ; PRJNA257286 , PRJNA388952 ; Graveley et al., 2011) and *Ilp3* in *D. melanogaster* using FlyBase release FB2022_04 ( GCA_000001215.4 ; Larkin et al., 2021; Gramates et al., 2022; Jenkins et al., 2022).

Invertebrate insulins function similarly to metazoan insulin-like growth factors and play a role in cell and organ growth (Jin Chan and Steiner 2000). In *Drosophila,* seven insulin-like peptides (Ilp1-Ilp7) have a two-chain structure similar to vertebrate insulin and interact with the sole insulin-like receptor, InR, to initiate the insulin signaling cascade (Brogiolo et al., 2001; Nassel and Broeck 2016). Like the *Ilp2* and *Ilp5* genes, the *Ilp3* gene is expressed in median neurosecretory cells (MNCs) in the brain (Ikeya et al., 2002). While the seven Ilps act redundantly with respect to promoting growth, they also have unique expression patterns and functions (Ikeya et al., 2002; Grönke et al., 2010). Ilp3 may act with the transcription factor dFOXO in a positive feedback loop to regulate Ilp2 and Ilp5 secretion from MNCs (Grönke et al., 2010). In female *Drosophila* , ablation of MNCs or knockout of *Ilp3* have been shown to reduce fecundity and remating rates (Grönke et al., 2010; Wigby et al., 2011). Knockout of *Ilp3* also results in sleep defects (Yamaguchi et al., 2022).

***Synteny***

The reference gene, *Ilp3* , occurs on chromosome 3L in *D. melanogaster* and is nested within *CG32052* , alongside *Insulin-like peptide 4* ( *Ilp4* ) (upstream) and *Insulin-like peptide 2* ( *Ilp2* ) (downstream). *Ilp3* is flanked further upstream by *L-2-hydroxyglutarate dehydrogenase* ( *L2HGDH )* and *CG43897* ( *CG43897* ) and further downstream by *Insulin-like peptide 1* ( *Ilp1* ) and *Z band alternatively spliced PDZ-motif protein 67* ( *Zasp67* ). The *tblastn* search of *D. melanogaster* Ilp3-PA (query) against the *D. ananassae* (GenBank Accession: GCA_000005115.1 ) Genome Assembly (database) placed the putative ortholog of *Ilp3* within scaffold scaffold_13337 ( CH902618.1 ) at locus LOC6507752 ( XP_001956273.1 )— with an E-value of 5e-08 and a percent identity of 31.13%. Furthermore, the putative ortholog is nested within LOC6507310 ( XP_014765147.1 ) alongside LOC6507751 ( XP_032309882.1 ; upstream) and LOC6507309 ( XP_001956274.1 ; downstream), which correspond to *CG32052* , *Ilp4* and *Ilp2* in *D. melanogaster* (E-value: 0.0, 3e-31 and 2e-27; identity: 86.67%, 51.52% and 46.79%, respectively, as determined by *blastp* ; Figure 1A, Altschul et al., 1990). The putative ortholog is flanked further upstream by LOC6507750 ( XP_001956268.3 ) and LOC6507311 ( XP_032310388.1 ), that nests LOC6502822 ( XP_001956270.2 ); which correspond to *L2HGDH* , *CG43897 ,* and *Ilp5* in *D. melanogaster* (E-value: 2e-111, 0.0 and 9e-09; identity: 68.06, 69.28% and 39.51%, respectively, as determined by *blastp* ). The putative ortholog of *Ilp3* is flanked downstream by LOC6507308 ( XP_001956275.2 ) and LOC6507753 ( XP_014765448.1 ), which correspond to *Ilp1* and *Zasp67* in *D. melanogaster* (E-value: 6e-35 and 0.0; identity: 52.46% and 71.34%, respectively, as determined by *blastp* ). The putative ortholog assignment for *Ilp3* in *D. ananassae* is supported by the following evidence: The genes surrounding the *Ilp3* ortholog are orthologous to the genes at the same locus in *D. melanogaster* and local synteny is completely conserved, supported by E-values and percent identities, so we conclude that LOC6507752 contains the correct ortholog of *Ilp3* in *D. ananassae* ( Figure 1A ).

***Protein Model***

Consistent with the *blastp* search result which shows 51.52% identity between *D. melanogaster* Ilp3-PA and the *D. ananassae* gene model, the dot plot features a few minor gaps along the diagonal, indicating significant conservation between the two protein sequences. *Ilp3* in *D. ananassae* has one protein-coding isoforms (Ilp3-PA; Figure 1B ). Isoform (Ilp3-PA) contains two CDSs. Relative to the ortholog in *D. melanogaster* , the CDS number and isoform count are conserved. The sequence of Ilp3-PA in *D. ananassae* has 54.74% identity (E-value: 8e-31) with the protein-coding isoform Ilp3-PA in *D. melanogaster* , as determined by *blastp* ( Figure 1C ). Coordinates of this curated gene model are stored by NCBI at GenBank/BankIt (accession BK064566 ). These data are also archived in the CaltechDATA repository (see “Extended Data” section below).

## Methods

Detailed methods including algorithms, database versions, and citations for the complete annotation process can be found in Rele et al. (2023). Briefly, students use the GEP instance of the UCSC Genome Browser v.435 ( https://gander.wustl.edu ; Kent WJ et al., 2002; Navarro Gonzalez et al., 2021) to examine the genomic neighborhood of their reference IIS gene in the *D. melanogaster* genome assembly (Aug. 2014; BDGP Release 6 + ISO1 MT/dm6). Students then retrieve the protein sequence for the *D. melanogaster* reference gene for a given isoform and run it using *tblastn* against their target *Drosophila* species genome assembly on the NCBI BLAST server ( https://blast.ncbi.nlm.nih.gov/Blast.cgi ; Altschul et al., 1990) to identify potential orthologs. To validate the potential ortholog, students compare the local genomic neighborhood of their potential ortholog with the genomic neighborhood of their reference gene in *D. melanogaster* . This local synteny analysis includes at minimum the two upstream and downstream genes relative to their putative ortholog. They also explore other sets of genomic evidence using multiple alignment tracks in the Genome Browser, including BLAT alignments of RefSeq Genes, Spaln alignment of *D. melanogaster* proteins, multiple gene prediction tracks (e.g., GeMoMa, Geneid, Augustus), and modENCODE RNA-Seq from the target species. Detailed explanation of how these lines of genomic evidenced are leveraged by students in gene model development are described in Rele et al. (2023). Genomic structure information (e.g., CDSs, intron-exon number and boundaries, number of isoforms) for the *D. melanogaster* reference gene is retrieved through the Gene Record Finder ( https://gander.wustl.edu/~wilson/dmelgenerecord/index.html ; Rele et al *.,* 2023). Approximate splice sites within the target gene are determined using *tblastn* using the CDSs from the *D. melanogaste* r reference gene. Coordinates of CDSs are then refined by examining aligned modENCODE RNA-Seq data, and by applying paradigms of molecular biology such as identifying canonical splice site sequences and ensuring the maintenance of an open reading frame across hypothesized splice sites. Students then confirm the biological validity of their target gene model using the Gene Model Checker ( https://gander.wustl.edu/~wilson/dmelgenerecord/index.html ; Rele et al., 2023), which compares the structure and translated sequence from their hypothesized target gene model against the *D. melanogaster* reference gene model. At least two independent models for a gene are generated by students under mentorship of their faculty course instructors. Those models are then reconciled by a third independent researcher mentored by the project leaders to produce the final model. Note: comparison of 5' and 3' UTR sequence information is not included in this GEP CURE protocol.

## Data Availability

Description: Zip file containing FASTA, PEP, and GFF of the model. Resource Type: Model. DOI: https://doi.org/10.22002/cajqt-ja897
